# Modelling in Synthesis and Optimization of Active Vaccinal Components

**DOI:** 10.3390/nano11113001

**Published:** 2021-11-08

**Authors:** Oana-Constantina Margin, Eva-Henrietta Dulf, Teodora Mocan, Lucian Mocan

**Affiliations:** 1 Department of Automation, Faculty of Automation and Computer Science, Technical University of Cluj-Napoca, Str. Memorandumului 28, 400114 Cluj-Napoca, Romania; Margin.Va.Oana@student.utcluj.ro; 2 Department of Physiology, Iuliu Hațieganu University of Medicine and Pharmacy, 400000 Cluj-Napoca, Romania; teodora.mocan@umfcluj.ro; 3 Nanomedicine Department, Regional Institute of Gastroenterology and Hepatology, 400162 Cluj-Napoca, Romania; 4 Department of Surgery, 3-rd Surgery Clinic, Iuliu Hatieganu University of Medicine and Pharmacy, 400000 Cluj-Napoca, Romania; lucian.mocan@umfcluj.ro

**Keywords:** QSAR, ALO, ANFIS, watershed segmentation, nanomaterials vaccine, anticancer physiology, image processing

## Abstract

Cancer is the second leading cause of mortality worldwide, behind heart diseases, accounting for 10 million deaths each year. This study focusses on adenocarcinoma, which is a target of a number of anticancer therapies presently being tested in medical and pharmaceutical studies. The innovative study for a therapeutic vaccine comprises the investigation of gold nanoparticles and their influence on the immune response for the annihilation of cancer cells. The model is intended to be realized using Quantitative-Structure Activity Relationship (QSAR) methods, explicitly artificial neural networks combined with fuzzy rules, to enhance automated properties of neural nets with human perception characteristics. Image processing techniques such as morphological transformations and watershed segmentation are used to extract and calculate certain molecular characteristics from hyperspectral images. The quantification of single-cell properties is one of the key resolutions, representing the treatment efficiency in therapy of colon and rectum cancerous conditions. This was accomplished by using manually counted cells as a reference point for comparing segmentation results. The early findings acquired are conclusive for further study; thus, the extracted features will be used in the feature optimization process first, followed by neural network building of the required model.

## 1. Introduction

Nowadays, detection, treatment, and also prevention of cancer is the main issue humans are dealing with, just because cancer involves a large group of symptoms and manifestations determined by even more combinations of initial triggers with variations of organism responses. Cancer is the general term used to define a great variety of health disorders that can affect any part of the organism. It develops from the alteration of natural body cells into cancerous, abnormal cells until they create a tumor. The cells start their transformation in the presence of external factors, such as chemicals, infections, or radiation. The process is extremely fast, and the multiplication of cells is uncontrollable, making it even harder to treat, because in most cases it is not detected in the incipient phase. Further, in this study, adenocarcinoma will be discussed, the main subject of multiple anticancer treatments that are currently developing in medicine and pharmacy study trials. Adenocarcinoma is a cancer type that is initialized in the glands, where fluids and mucus are produced for normal functioning, and spreads to other organs [1]. The most concerned organs are breasts, lungs, colon and rectum, and prostate, which are also situated on the first four positions in the top occurrences of cancer diagnoses, respectively. Moreover, lung and colorectal cancer are the most frequent causes of cancer death [2], according to the World Health Organization.

The starting point for the development of a treatment was guided by a research team from the “Iuliu Hațieganu” Faculty of Medicine and Pharmacy [3]. The proposed treatment implies the creation of an anticancer vaccine that has the main goal of triggering the immune response to destroy unwanted cancerous cells.

There exist two types of cancer vaccines, namely preventive and therapeutic. Both of them target the immune system in order to distribute into the body the description of cancerous cells, and furthermore to train the system to eradicate them. In the case of cancer, the problem is more complex because cancer cells are similar to healthy cells, and each tumor has its own specificities from one person to the other [4]. Fortunately, since the medicine has evolved simultaneously with the technology, the potential of therapeutic cancer vaccines has advance, being able to differentiate cancer and normal cells.

The therapeutic vaccine from [3] employs nanoparticles of gold to associate suitable antitumor antigens for activating the immune system. The approach offered optimistic preliminary results that need to be further expanded, this study being an innovation. To forecast cell activity under the impact of a certain therapy, the main objective of the research is to determine the mathematical model of the vaccine; however, the most important goal that must still be achieved is to process cell images, then extract and measure numerical information about vaccine efficiency in laboratory experiments. As such, in the current work are emphasized the chosen and applied image processing techniques. Subsequently, the extracted data will naturally show the relationship between vaccine components and molecule structures.

Since the general aim of the research is the optimization of structure based on nanostructure–effects correlation, computational models, especially those based on Quantitative-Structure Activity Relationship (QSAR) approaches, have been successfully applied to Computer Aided Molecular Design (CAMD) problems that involve small organic molecules. The current task employs this computational technique to compute the nanostructure effects by linking this activity to a set of compositional and structural properties. The defining or coding of the chemical structure using a variety of molecular descriptors, such as constitutional, topological, thermodynamic, functional groups, quantum mechanical, geometrical, and many others, is a critical step in this research. The models that are created will be used in both prediction and optimization stages.

Training data necessary to develop a model have their origins in controlled experiments, more specifically the in vitro (meaning the study is executed outside of a living organism) experiments and are used to determine properties or relations between cancerous cells and the compound mixture proposed for treatment. The experiments conducted in laboratory must be centralized, and significant data should be extracted in order to create the molecular descriptors data set that will further determine the model. Hence, all microscopic images depicting cell activity are acquired and have to be processed to obtain important conclusions. Because this procedure would be difficult for a person to do, and the time required for this task would be certainly higher, the best suited software algorithms can be trained to facilitate the work while offering better results, since human perception can be easily influenced by subjectivity, experience, and diverse personal conditions. In order to decrease the human errors as much as it is possible, hyperspectral images are subjected to specially designed algorithms to segment cell regions and determine each cell’s characteristics. These cell characteristics will be employed in the set of descriptors necessary for the model.

The classification of QSAR methods implies two main categories: one regarding the dimensionality, and one related to the linearity/non-linearity approach, as stated in [5,6]. On the other hand, one traditional assumption states that biological activity can be modeled using linear equations using a regressive combination of descriptors and coefficients. Linear methods comprise linear regression, partial least-squares, and principal component analysis. However, nature can be only approximate using linearity. Non-linear QSAR models avoid the limitations introduced by linear ones and represent a better fit for real biochemical relationships encountered. These include machine learning techniques such as Bayesian neural nets, k-nearest neighbors, and artificial neural networks (ANN), sometimes combined with fuzzy logic in adaptive neuro-fuzzy inference systems (ANFIS) [5].

For establishing the limit value where the treatment may start to bring desired results, the literature specifies point-of-departure (POD) quantity. In [7], two QSAR models are used for predicting the POD values in order to ensure low human health risk level. For developing a QSAR model, many intermediary steps should be followed. The first step after collecting data requires data pre-processing. This means that the data sets are prepared using different transformations, including normalization, noise and redundancy elimination, data cleaning or reduction, discretization, or others [5].

The second important step would be the split of the data set into training and test sets, but they must cover all the information that was gathered, i.e., the entire data set. The training set is used for model development, whilst the test set is used to evaluate to what extent model predicting is effective and reliable. There are many ways in which we can consider the splitting [8], each one having different performance levels. Goodness-of-fit assesses how well the model predicts the training set. For internal validation, there is cross-validation, which is also used in [9]. Moreover, as external validation, the whole set is divided in two specific parts, for most of the cases using a proportion such as 80–20% (or 70–30%) training and testing sets, respectively. In this case the objective is how well the model predicts the test set. Each approach has benefits and downsides; therefore, several factors must be taken into account to prevent too optimistic results: data variety, statistical methodologies, sample sizes, and research goals. One can see problems in the model structure by comparing the outcomes of the three distinct techniques.

The next step for the development phase is the selection of molecular descriptors used. Depending on the information stored by these descriptors, the dimensionality of the model is chosen. Additionally, because the data set can contain numerous features, only the most suitable ones are selected to influence the model. There are several methods that could be used in this direction, namely filtering techniques, heuristic methods, or recursive methods. More details about pros and cons are described further. Models are then constructed using statistical methods or machine learning algorithms. The models are finally tested with previously described methods, such that ultimately an optimization procedure will offer a satisfactory prediction model.

Some of the parameters describing the molecules must be determined based on experimental images provided by the research team from Medicine and Pharmacy University. In this case, image processing is another aspect that needs to be reached. MATLAB also provides an image processing toolbox including functions for analyzing hyperspectral images. Multiple methods can be used to analyze images containing viable cells. Further are presented several approaches regarding image processing from the medical field, with emphasis on viable cell segmentation and relevant information related to digital images.

In order to extract accurate cell features from hyperspectral images, one important aspect is the recognition of the cell contour. In [10] a complete process for contour identification of cells is subjected.

Another study [11] suggests a machine learning approach for recognition of cell contours and protein classification. Their graphic method is based on feature detection and extraction, although tested in two variants: field-level and cell-level specifics. The method is similar to [10], but this one guarantees high accuracy, even for condensed cells.

Machine learning and also deep learning techniques have gained attention lately, because compared to classic methods, these are more robust with accurate results, although human intervention is minimal. There are two approaches for machine learning as presented in [12], namely supervised and unsupervised learning algorithms. As described in [13], researchers managed to determine and train an unsupervised model that accurately detects relevant areas and cell characteristics on fluorescent neural images.

Segmentation of cell images is the milestone for all studies and quantification problems, because data analyses require information about individual cells or cells grouped as a whole, but the aim is to differentiate them from the background. A deep learning approach has been developed also in [14] for accurate classification and detection of constituent parts from cell images.

Another possible approach to image segmentation would be application of watershed transform. In [15], image analysis is performed on yeast cells. The main principle is that each image will be transferred to a gradient image from which a selection of local minima is chosen. This selection represents the starting points for a so called “flooding” action, if taking the analogy with a topographic area.

In order to analyze how effective a drug treatment is, authors in [16] propose several clearly established steps to create a characteristic profile essential in many identification studies. The first step implies brightness adjustment, segmentation, and extraction of features. For proper segmentation, even illumination has a major impact, since cells in darker areas can easily corrupt the measurements. The correction can be computed for the entire set of images, for each image separately, or with respect to a reference. From the three main methods that can be addressed, computing a correction function for the whole set has better outcomes when the interest is directed to quantity. The segmentation might be performed classically through a series of procedures (thresholding, edge detection, watershed transformation) or using a machine learning approach.

Before choosing the model approach that is consistent with the experiment, all advantages and drawbacks must be considered for each method, such that a machine learning technique has improved performances regarding image segmentation for different varieties of cells, but for training stages, a lot of manual settings are necessary, i.e., pixel labels. On the other hand, model-based approaches have great performance levels for darkfield hyperspectral images with the downside of manually choosing each step of the algorithm carefully in order to gain the most optimal segmentation outcomes based on visual determinations/approximations.

Getting back to development of QSAR models, as stated earlier in this study, due to molecular relationships that are highly nonlinear, we must focus on nonlinear model approaches. A comparison between k-nearest neighbor (k-NN) and support vector machine (SVM) regression methods are presented in [17] for a class of cancer inhibitors. A support vector machine method is developed in [17] as a linear regression method by considering the features computed for each compound as inputs to an approximated linear function for which the output is the measured activity.

Artificial neural network is one of the other QSAR modelling methods; however, because it depends on parameters used to achieve a higher precision, it can easily end up in local optima. As stated in [10], a better approach would be to combine ANN with the fuzzy logic rule set, resulting in the ANFIS model (adaptive neuro-fuzzy inference systems). The subjective human element is added to the ANN’s machine learning process through fuzzy logic system. Takagi and Surgeon [18] established the first fuzzy technique, which consisted of a set of fuzzy rules for generating a nonlinear association of inputs with outputs in the form of IF premise THEN consequent.

The ANFIS architecture is a five-layer neural network with the same function for each layer’s neurons, all of which are part of the fuzzy inference process: determining fuzzy values, fuzzy rule firing strength, firing strength normalization, combine premises with consequents, predict, and final output. The weight parameters between layers are used to accomplish optimization.

The data is fed into the net, and on forward pass, the resulting parameters are generated using least squares estimation [19], and once the deviation is identified, the premise factors are settled using backpropagation from output to input by gradient descent.

Since the usage of genetic algorithm (GA) or the PCA introduces limitations in terms of convergence, data precision, and time costs, the approach followed in [9] uses the antlion optimizer (ALO) [20], a novel idea inspired by the natural hunting process of antlions and ants.

Even if the antlion optimization algorithm developed in [20] presents efficient solutions, in many diverse optimization problems, the method was not evaluated in terms of runtime. The cause of this drawback is mainly the random walk described by ants. As a solution, an improved algorithm was advanced in [21], namely “tournament antlion optimization algorithm” (TALO). Through the research [21], the analysis between ALO and TALO indicated superior results in the improved method considering multiple references such as mean deviation, best/worst cost, time to find global optimum, and precision.

This optimization algorithm’s main objective is to improve the final accuracy and adjust the amount of satisfactory characteristics. The ANFIS model uses the generated collection of characteristics as input descriptors to anticipate biological processes.

The final product of this current research step is a user-friendly application for determining cell characteristics from acquired images. The image processing software will benefit directly the biomedical specialists or the engineers that will have to use the extracted data for model development, but indirectly all patients who will be able to receive the tested treatment in safe and efficient doses.

## 2. Materials and Methods

In this work, image processing has a significant influence on extracting and measuring numerical information on vaccine efficacy, which is then employed as parameters in the model design. Depending on their importance for the intended model, these characteristics might include cell brightness, surface, form, or counting. Because the final accuracy is directly connected to the consistency of information processed, data sets are the most essential factor to consider while building a predictive model. Using image processing technologies, numerous characteristics may be retrieved from the experimental photos given by the medical research team. There are a variety of approaches that may be employed, ranging from conventional to deep-learning techniques, all of which produce good cell segmentation results.

The most important goal is to achieve cell segmentation, which entails dividing a microscopic snapshot into areas that represent individual cells and distinguish them from the background. Then, one major task is to collect information on single cells or groups of cells determined. Following the collection of data, molecular descriptors are calculated to identify the set of important characteristics that best predict biological activity values. The optimization process begins with the selection of these characteristics. The work’s uniqueness is in the use of appropriate image processing techniques that best meet the requirements before moving on to model creation. The given pictures are pre-processed for quality improvement, morphologically modified to produce smoothness and more regular forms, and cells are labeled using watershed segmentation on enforced regional minimums so that features may be retrieved efficaciously. The next step is then employment of ANFIS optimization algorithm.

The current study’s subsequent parts discuss the methodologies and algorithms used for image segmentation and model creation, as well as some findings obtained related to the processing of darkfield hyperspectral image data.

### 2.1. Image Processing

The importance of image analysis comes from the fact that biomedical images enclose a wealth of entities and shapes, which may transmit information about fundamental mechanisms in biology.

With a view to obtaining the desired results, specifically as a first phase, image segmentation followed by extraction and quantification of entities, depending on applied dose of medication, the current developed algorithm is based on the classical image processing approach. Segmentation may be accomplished using a variety of methods, the most popular of which is edge detection, which is widely employed in cell determination. However, because the forms in this task are very uneven and the cells are packed, contours cannot be detected effectively using edge detection or machine learning approaches if the training set is not sufficiently vast. Watershed segmentation, on the other hand, has been effectively used for a variety of entity separation issues.

The inputs to the current algorithm are images in raw form as they are acquisitioned from the microscope. The outputs are features extracted from those images. One major step for achieving this is through segmentation.

#### 2.1.1. Image Segmentation

For individual quantification of each cell, edges of cells must be detected in a process known as cell segmentation. Hence, the procedure used to establish which pixel is part of which specific cell is image segmentation.

Images are partitioned into fundamental components or regions, and the stopping criterion should be met when the entities we are interested in have been separated. For example, in applications like the current one, where we want to confirm the presence of intercellular absorption, there is no purpose in leading segmentation further than the level of detail needed to classify each element into an individual category. When talking about image processing, segmentation of unusual images is the most challenging task, which is why substantial attention must be directed to enhancing the accuracy that concludes the eventual success or failure of automated analysis procedures.

Image intensity values underlie the segmentation algorithms through either of two properties: discontinuity and similarity. Firstly, when approaching discontinuity, the partition is based on steep changes in intensity, such as edges and contour detection. The main procedure regarding the second category is based on dividing an image into zones that are analogous according to a set of predefined conditions. The main methods employed here are thresholding and region detection.

The segmented objects are usually labeled as foreground while the rest of the image is the background. It should be mentioned that normally there is not just one accurate segmentation, no matter the processed image. The types of entity or areas we are concerned with identifying determine the proper segmentation of the image.

When we try to fragment images, there are three elementary features that we can utilize: color, texture, and motion. Having the current purpose, color is the most straightforward and easy way of discerning between objects and background.

#### 2.1.2. Watershed Transform

The watershed transform is the core method used for multiple developed techniques for image processing, starting from traditional methods with a manually selected flow of functions, through to deep neural networks that newly advanced, most of them reasoned on watershed type transformations. Therefore, the main reason for the traditional approach having been chosen for this particular project is the fact that all pictures received have their uncommon specificity; the research is still in progress, such that the set until present time cannot support neural network training. Moreover, we are not interested in classifying tissue or cells; we need to measure intensity-based features. This approach is also favored to the detriment of others, because it is efficient in cases of objects that are strongly touching, one of the greatest challenges in cases of image processing and entity identification.

**Figure 1:**
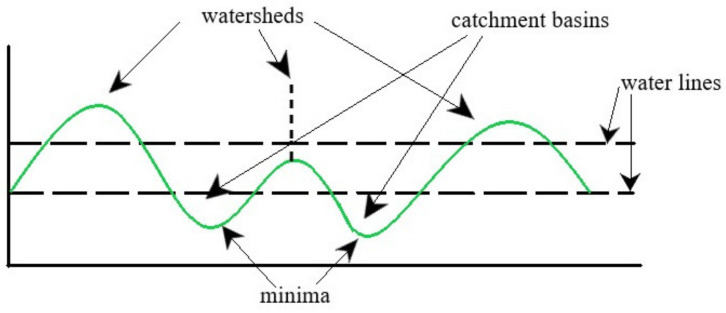
Watersheds and basins illustration.

Considering a 2D image having one single channel, watershed segmentation treats it as a topological surface in which the location is specified by the x, and y image array indices and the pixel intensity values are interpreted as heights. The principle underpinning the algorithm is derived from the analogy with the simple natural phenomenon of rain being drained toward the local minimum point when influenced by gravity, forming a catchment basin. For reference, Figure 1 clarifies the described aspects. A catchment basin represents the region naturally created to collect any rainfall in the same minimum point. When speaking about digital images, the catchment basin is made up of a cluster of associated pixels.

While keeping with the topography analogy, the local maximum point where rain is likely to fall either towards one catchment basin or to another are similar to the peak of a mountain or to dams that prevent water from dissipating between adjacent basins, and is dividing one catchment basin from another, these delimiting lines are called watershed ridges, from which the method’s name comes. The initial image we are trying to segment is transformed to an image where the objects concerned resemble the catchment basins detected by the watershed transform [22].

**Figure 2:**
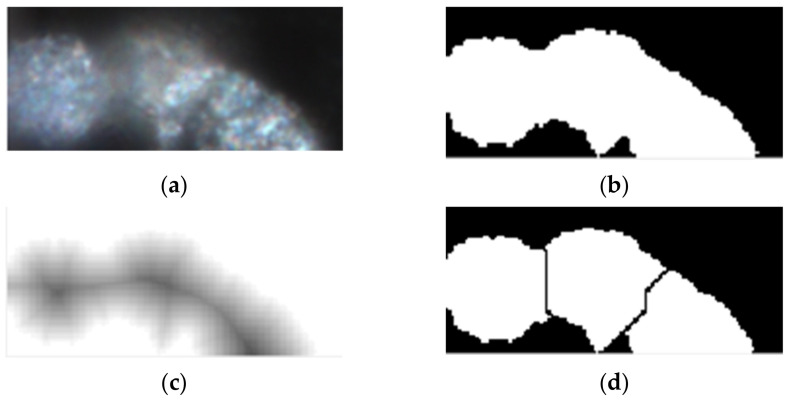
(**a**) Original RGB image; (**b**) thresholded image; (**c**) distance transform; (**d**) segmented regions/cells.

The original image (Figure 2a) includes three different cells that have absorbed treatment compounds. After being transformed using thresholding (Figure 2b), a function of the original image must be derived from it using distance transform (Figure 2c), in order to determine the catchment basins required in a watershed. In an ideal scenario, the watershed ridges calculated would lie along the object edges, as in Figure 2d. 

#### 2.1.3. Pixels and Histograms

Usually, pixels are image elements that hold the color or intensity in small points representative of colored light samples from the scene. Best results in any image processing assignment are achieved after utilizing pre-processing techniques on pixels for enhancing image quality and visual descriptions.

The histogram of an image is a function that indicates how many pixels have a certain intensity level. In general, the number of color or gray levels is 255 (one pixel is represented on 1 byte), represented as values on the *x*-axis of the plot. The *y*-axis consists of a frequency vector depending on how many pixels incorporate each value encountered.

When analyzing a histogram of diverse types of images, one can observe that an image has multiple intensity levels, and it presents two local maxima. Using this characteristic, we can infer a suitable threshold that can be used to segment the initial image, by choosing the threshold as the local minimum between the two maximum points. Thus, the pixels with lower intensity levels than the designated limit could be considered part of the background, while the ones with higher values are the constituent pixels of the objects, the foreground. Contrast tunings on the image can also be envisioned by employing histograms.

**Figure 3:**
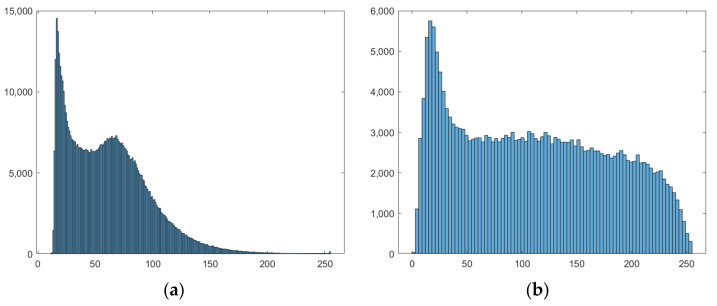
(**a**) Original image histogram; (**b**) adaptive histogram equalization applied on (**a**).

When the histogram of an image has a wide distribution, while it presents also extremely skewed local regions, the contrast in these regions should be improved. However, it would be more efficient to use the adaptive histogram equalization (Figure 3) rather than employing a global equalization that would offer a general brightness transformation and cannot properly adjust the contrast local regions situated in the dark background, for example [23].

The usage of the word “adaptive” emphasizes that specific regions from an image are addressed in a distinct way based on regional characteristics, and further that the method is expanded to the contrast-limited technique. The problem of noise intensification can appear when the contrast is enhanced too much after employing this procedure, because any details in the region would be intensified particularly, including noise fragments and artifacts, having a great receptiveness to unintended and out of hand variations in the radiance. In order to avoid this, contrast limiting can be successfully used, such that by setting a global limit from the interval [0, 1], the contrast enhancement for all areas is kept under control [22].

#### 2.1.4. Otsu Thresholding

Global thresholding is best applied on images where the objects have a similar intensity and color while the background is of opposite intensity, not having a great variation of intensities with very dissipated pixels. In this case the setback of discontinuity of objects can appear because luminous pixels do not have to be connected in space. Typically, the threshold method can be applied successfully when the distribution on the histogram plot is evidently bimodal, by choosing a threshold from the local minimum between the two maxima. However, when the image has a diverse range of values and multiple peaks, the threshold selection is more problematic. Otsu’s method can be applied on grayscale images, and it automatically computes the threshold based on statistical variance of the black and white pixels. That is, the foreground and background pixels are determined by minimizing the variance class. The class variance is defined by Relation (1).

(1) $$\sigma_{B}^{2}\left(k\right)=\frac{{\left[(m_{G}P_{1}\left(k\right)-m\left(k\right)\right]}^{2}\;}{P_{1}\left(k\right)\left[1-P_{1}\left(k\right)\right]},\;$$

The term *m_G_* is the mean intensity for the whole image, and the variable terms are *m*_1_ (the mean intensity between levels starting from first until *k*th one) and *P*_1_, the occurring probability of pixels set until the *k*th level.

#### 2.1.5. Morphological Operations

For achieving the best results, prior to thresholding and segmentation transformations we have to ensure that the images have the necessary qualities and fulfill the structural conditions for achieving the main goal. Morphological operations are mathematical and computational techniques developed with the aim of structural changes in image processing such that elements from captures are effortlessly detected to extract meaningful data. The inputs and outputs from morphological approached methods are images defined by two-dimensional arrays.

The operations selected as relevant and used in this current work are opening, closing, structuring element decomposition, and area open. These approaches are directed towards objects in binary images to enhance shapes and edges by using structuring elements. The fundamental processing functions are dilation and erosion, which can be used together in different combination to create the opening and closing operations. Each object is defined by pixel connectivity, which is characterized by pixel neighborhoods.

Coming back to dilation and erosion operations, which are the most important functions that morphological transformations operate upon, as the naming is directly showing, dilation is the process in which the objects in an image are superimposed with structuring elements of any shape we desire, according to the set operation from (2), where A is the binary image, and B is the structuring element.

(2) A⊕B={z|B^z∩A≠∅},

The shape of the structuring element is chosen to best fit the object shapes, in two-dimensional space, and usually the overall dimension is small when compared with the image we desire to apply it on.

Similarly, erosion is the inverse operation to dilation and resembles shrinking of the object, also using the structural elements. The relation for characterizing this can be found using Relation (3). By the use of this, insignificant secluded features are eliminated.

(3) $$A⊖B=\{z|{\left(B\right)}_{z}\subseteq A\}$$

Therefore, the interest is determined by the combination of the two (Relation (4)); explicitly, an erosion subsequent by a dilation is equal to opening an image.

(4) $$A\circ B=\left(A⊖B\right)⊕B,$$

(5) $$A\cdot B=\left(A⊕B\right)⊖B,\;$$

Quite the opposite is the closing operation, constituted by the sequence dilation–erosion (Relation (5)). The understanding is straightforward when thinking of unregular shapes. These operations enhance edge discontinuity, a very common procedure for cell and cytoplasm determination. Putting it simply, opening is the process of eliminating unimportant, outlying objects from the foreground and smoothening the edges, while closing is used to fill small hollows in the foreground.

### 2.2. Feature Extraction

The goal is usually to process the picture in such a manner that it or its attributes can be effectively represented and retrieved in a condensed form that can be recognized and classified afterwards. Internal and external representations are the most common. Internal refers to the pixels that make up an area, for example texture and intensities. External refers to the pixels determining the limits of an area, for instance shape-based representations. In some cases, a shape study/identification approach is not enforced to produce an accurate, regenerated shape depiction. In order to gain insights about cell characteristics, the purpose is to extract meaningful and straightforward features, such that their number can be kept as limited as possible to not harden the whole process. For this purpose, we have to identify beforehand all important descriptors that can be extracted and offer sufficient data for achieving the project goal. In Table 1, one can find a list of the most frequent single-parameter approximate measurements that can be used as structural attributes in distinction and categorization basic procedures.

Note that several descriptors are computed with the help of the measured perimeter, total area of bounded foreground objects, and extreme points values, all measures effortlessly determined in practice. By combining variations of area and perimeter values, it results in determination of dimensionless changes. The circle and square shapes offer in general very significant reference values for these quantities.

### 2.3. ALO–ANFIS Algorithm

**Figure 4:**
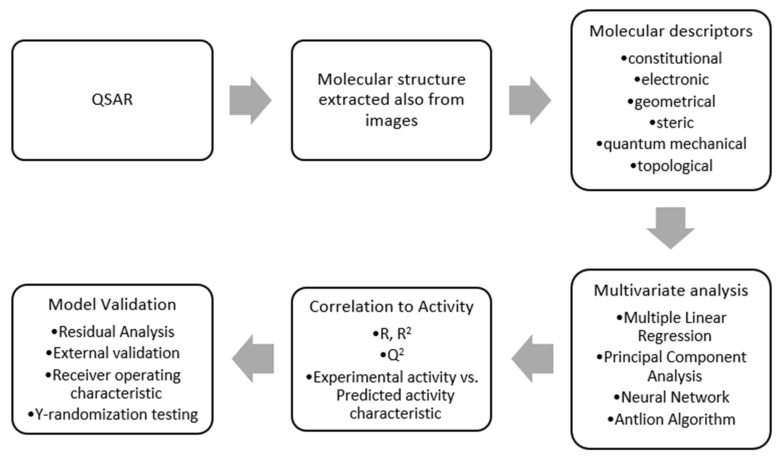
Workflow for chemical model development.

The adaptive neuro-fuzzy inference (ANFIS) model combines two efficient methods [24], thus gaining best characteristics to resolve complex regression problems such as biomedical QSAR models. Moreover, the gradient method employed by the learning rule is enhanced in this way to avoid local minima trapping and slow processing speeds. The workflow for achieving a predictive model is presented in Figure 4.

A fuzzy inference system is made up of five different functional blocks [15]. The rules suited for the inputs we have to give to the system are of type Takagi–Sugeno [18], each rule’s output being represented by a linear combination of input variables.

The nodes of an adaptive network mean that each of their outputs is dependent on the node’s parameters, and the learning rule defines how these parameters should be adjusted to minimize a specified error measure. The gradient descent and the chain rule, introduced by Werbos [25] in the 1970s, are the core learning rules of adaptive networks.

The initial step of the fuzzification process involves determining fuzzy values from inputs using membership functions (MFs). The MFs are markers of values belonging to a specific cluster, in which the data inputs are split into various categories in a low to high range, with a mean value and a deviation that indicate the degree of similarity between these values. The membership functions use a bell-shaped Gaussian function to map input values—x, y—based on which category they correspond to. Every data entry will be characterized by a membership value to a category, resulting in n × m nodes in this layer, where n denotes the number of members (inputs), and m denotes the number of categories.

Layer two’s firing strength is just a weight calculated using the previously fuzzified data; thus, each weight represents the strength of the corresponding rule from layer one. Layer three normalizes the weights, allowing each weight to be compared to the others, with the rule being that the greater the strength, the better.

The generated weights are joined with the input variables again in the fourth phase to produce the relevant function and output values, which are added up in the fifth and final layer to determine the projected activity. Forward pass and backpropagation are two strategies used in the learning process.

The impact of using multiple descriptors, which are widely employed in medicine and biological areas, is a disadvantage of the ANFIS approach. Precisely, the net’s complex nature would rise to the point where it might cause overfitting difficulties specified by the training parameters, lowering overall accuracy.

Given that the maximum accuracy is sought for every QSAR model, a careful selection of the most important descriptive characteristics that will define the model and, subsequently, the biological activity prediction is a must. As an optimal goal, using a selection of descriptors will increase performance of the algorithm employed by increasing the time effectiveness while decreasing computing costs.

As stated also previously in this work in more detail, the optimization is achieved through the antlion optimization algorithm (ALO), which is used for feature selection to input just the most valuable descriptors to the process. The strategy adopted is inspired by the chaotic walking path of ants [26] until they get trapped in an antlion pitfall. The two phases of the algorithm have together six steps that are iterated until the stopping criterion is encountered. Chaos is a deterministic dynamic process that is highly sensitive to its initial settings. Chaos is obviously unpredictable and random, yet it also has a bit of uniformity.

## 3. Results

All of the procedures and functions were written in the MATLAB programming environment and run on a 64-bit Windows system, with the Image Processing toolbox used for segmentation. The final graphical user interface was developed using the Design app package and the Application compiler. The MATLAB program is a great choice from the present point of view because it offers a wide library of built-in functions determining flexibility in combining different development domains, as well as simple syntax.

In the current section are presented the applied concepts and the obtained results. The scope of the implementation is descriptors extraction and creating the possibility of comparative analysis between different stages of applied treatment. The final product is the application that can be used equally by medical authorized persons working on the project or by other engineers that will be in charge of further model implementations.

The purpose came from the need of assessing drug behavior. For accomplishing this, the medical team that launched the research gathered the data set images using high performance darkfield microscopy with hyperspectral imaging (Cytoviva Inc., Cluj-Napoca, Romania) from laboratory experiments in controlled environments. The fluorescent component absorbed by cells was successfully captured in darkfield hyperspectral RGB images on dark backgrounds. Each group comprised of four hyperspectral images represented four different stages of cell viability depending on the quantity of drug administered.

One important thing that the study assessed is cell viability and proliferation ratio given by the quantity of the fluorescent compound that entered the cytoplasm and the degree of clustering effect after exposure to nanoparticles from the compound.

**Figure 5:**
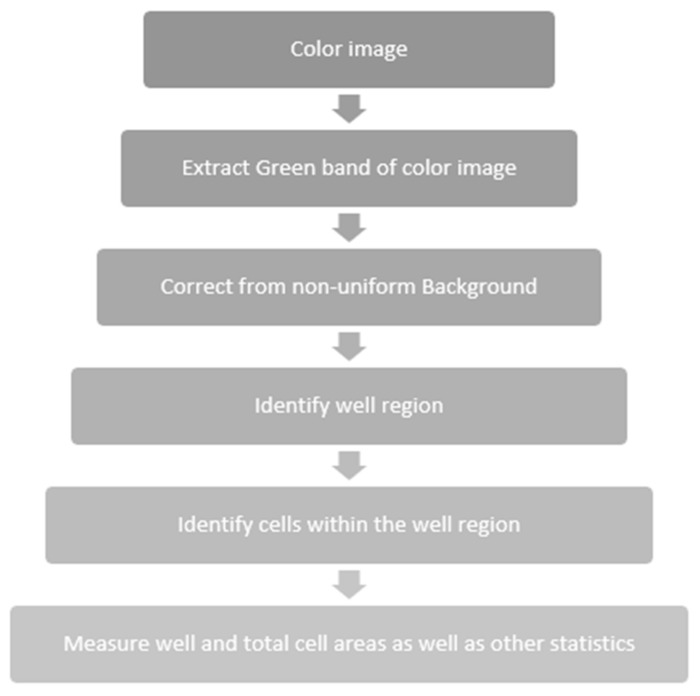
Processing workflow.

**Figure 6:**
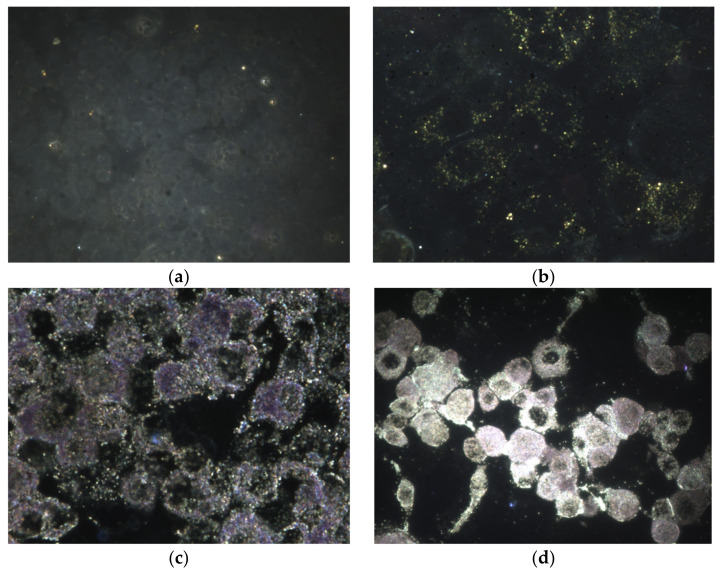
Cells (macrophages) exposed to various drug quantities: (**a**) Stage A—Control; (**b**) Stage B—12.5 μg/mL; (**c**) Stage C—25 μg/mL; (**d**) Stage D—50 μg/mL.

The workflow for processing hyperspectral images [27] after image acquisition is presented in Figure 5. In this research we were concerned about quantifying the total of green fluorescence incorporated in cells exposed to special experimental settings. The research team from the “Iuliu Hațieganu” University of Medicine and Pharmacy contributed the data set. Following a series of studies, some of their findings included darkfield hyperspectral image sets depicting the effect of therapy applied to cell cultures, especially the responsiveness of cells to various dosages (Figure 6).

Of particular relevance for this study is the contrast in pictures between stages with applied treatment and control phases, whose cell culture was untreated. An examination of white light reflection points coming from metal nanoparticle component as well as on the membrane was conducted to lead to preliminary findings that could be drawn in order to establish the existence of the bio-nanocomposite within the molecular region. To provide good performance and correct segmentation, each image passed through a number of pre-processing stages, such as noise suppression, contrast adjustment, and morphological opening–closing. These steps are presented in more detail in the further subsections.

### 3.1. Pre-Processing

A high-resolution digital camera was used to take the images. Each image was processed individually because it was passed as a parameter to the *cell_segmentation* function. Before making any other enhancement operations, all images were dimensioned at a predefined number of pixels in order to reduce processing time and to keep the same proportions, which is also a very important step for comparing structures and quantities in an image.

Images were defined by a set of three arrays, each representing the red, green, and blue channels that constituted the image. Each cell from the three arrays represented a pixel from the image, and the value retrieved from it was the intensity of the respective color in the channel.

**Figure 7:**
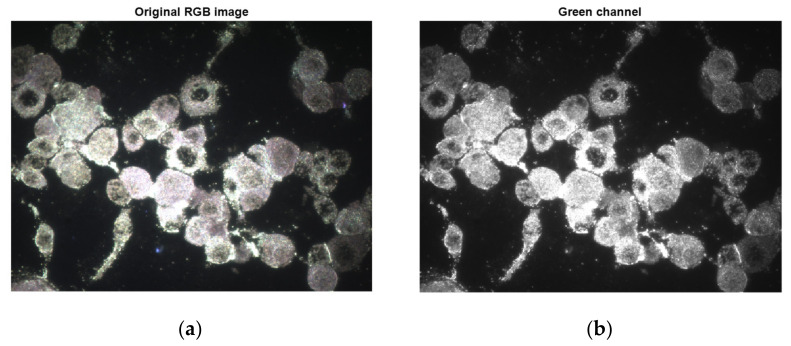
Green channel extraction: (**a**) original RGB image; (**b**) green channel.

Since we were interested in measuring fluorescent objects, firstly the green band from the RGB representation was extracted, and thereafter the image engaged in the process with just one channel (Figure 7).

**Figure 8:**
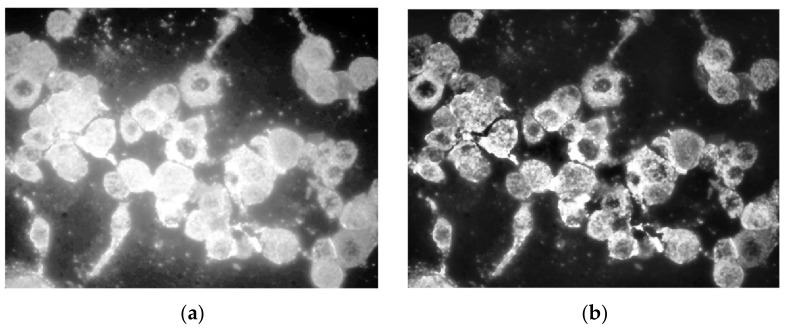
(**a**) Global contrast equalization; (**b**) adaptive contrast equalization.

High-quality laboratory equipment produces pictures with maximum intensity, eliminating the need for brightness uniformization. Contrast adjustment, on the other hand, is a useful enhancement primarily for developer analysis, as the conversion from color image results in a faded image. To enhance contrast equalization in the suggested implementation, an adaptive approach was employed, namely *adapthisteq*, with a low limit value for enhancing details while keeping unimportant intensities as noise. As shown in Figure 8, this function has a significant advantage over global enhancement techniques in that it treats limited sections of the picture depending on histogram evaluation, preventing noise amplification [28,29].

Before adjusting the contrast, a median filter should be used to reduce the noise that may have entered the original image. In contrast to mean filtering, a median filter produces more effective results while maintaining edges by finding a statistical median of nearby pixels.

**Figure 9:**
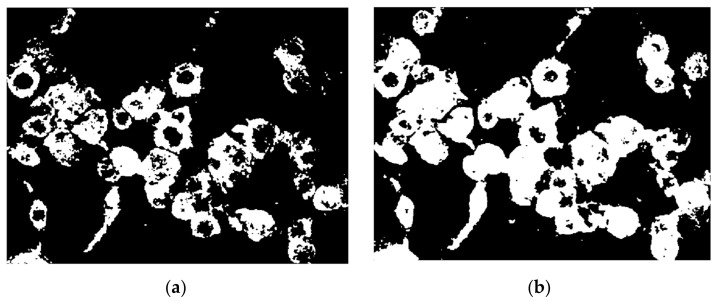
Binary conversion using (**a**) global threshold, and (**b**) Otsu threshold.

The picture is binarized further using a global threshold established by the Otsu technique. The function that has the greatest results is *imbinarize*, newer than *im2bw*, and in combination with the Otsu determined threshold, all fluorescent cells are fully converted, not just small portions. A comparison between the application of the global determined threshold and the specialized one is depicted in Figure 9.

**Figure 10:**
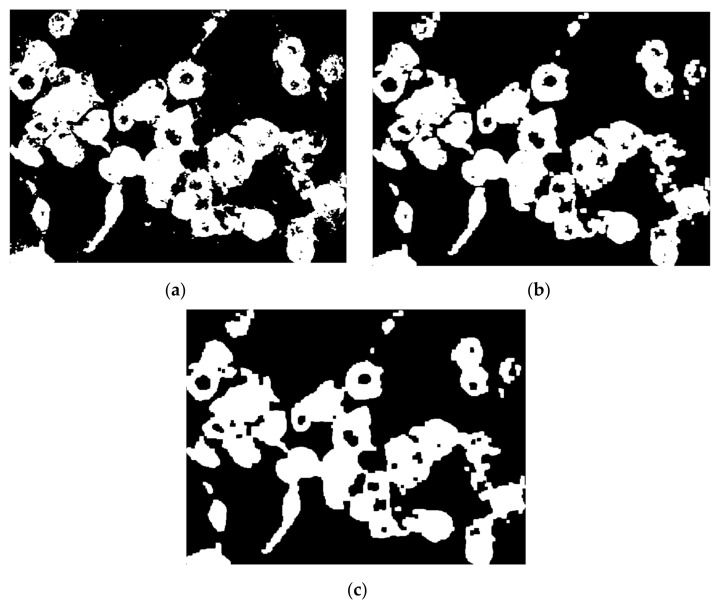
(**a**) Binary image; (**b**) morphological open on (**a**); (**c**) morphological close on (**b**).

Further, instead of smoothing the edges using a kernel filter, the morphological operations of opening and closing were engaged in the process to enhance smoothness of all cells detected. As presented in a previous section, the opening transformation (Figure 10b) was “cutting” all extremities that came out of the contour, and the closing filled the margins to make them continuous (Figure 10c). On the other hand, by using the kernel filter, the entire image was blurred to create the smooth effect, but in this way the overall resolution quality was lost, including the details, because this would be applied before converting to binary image. Both morphological transformations were performed by means of disc-shaped structuring elements of radius 3.

Relatively small stains that are inconclusive in terms of cell segmentation can be treated as noise and eliminated, allowing the number of cells calculated from the segmented picture to remain unaffected. Correspondingly, *bwareaopen* may be used to remove minor disturbances within the cell area, first removing white foreground regions from the dark background.

Then, since cells are in the front, small black patches inside molecular regions are created by complementing and removed. In this case, all regions smaller than a selected parameter of 50 pixels were eliminated from the picture.

### 3.2. Segmentation

The watershed segmentation is based on the idea that each item we wish to recognize should be a catchment basin, which means we need to use a gradient to modify the picture and then pick a set of local minima.

**Figure 11:**
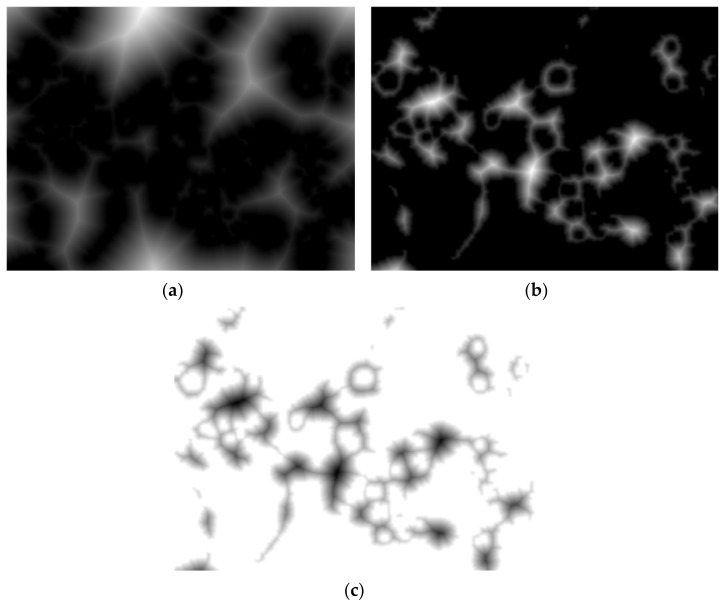
(**a**) Euclidean distance transform, (**b**) EDT on complement image, (**c**) negated (**b**).

The Euclidean distance transform (EDT) provides a converted image from the binary one, where each pixel is determined by Euclidean distance from the closest pixel different than 0 (Figure 11a), meaning that we have to compute quite the opposite because foreground pixels from the binary image will become the darker ones. To overcome this inconvenience, we can complement the initial image and then apply the transform (Figure 11b). However, we still need each object to become a catchment basin (see Section 2 for reference) so by negating, we turn the bright areas into this (Figure 11c).

**Figure 12:**
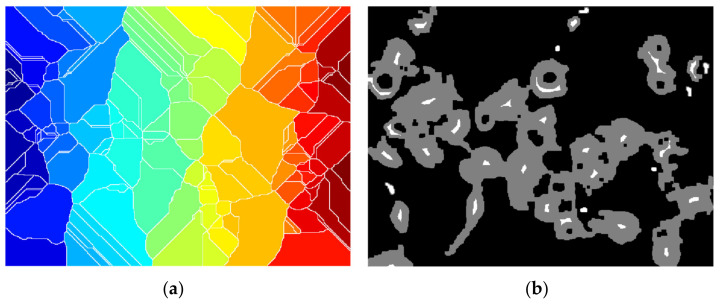

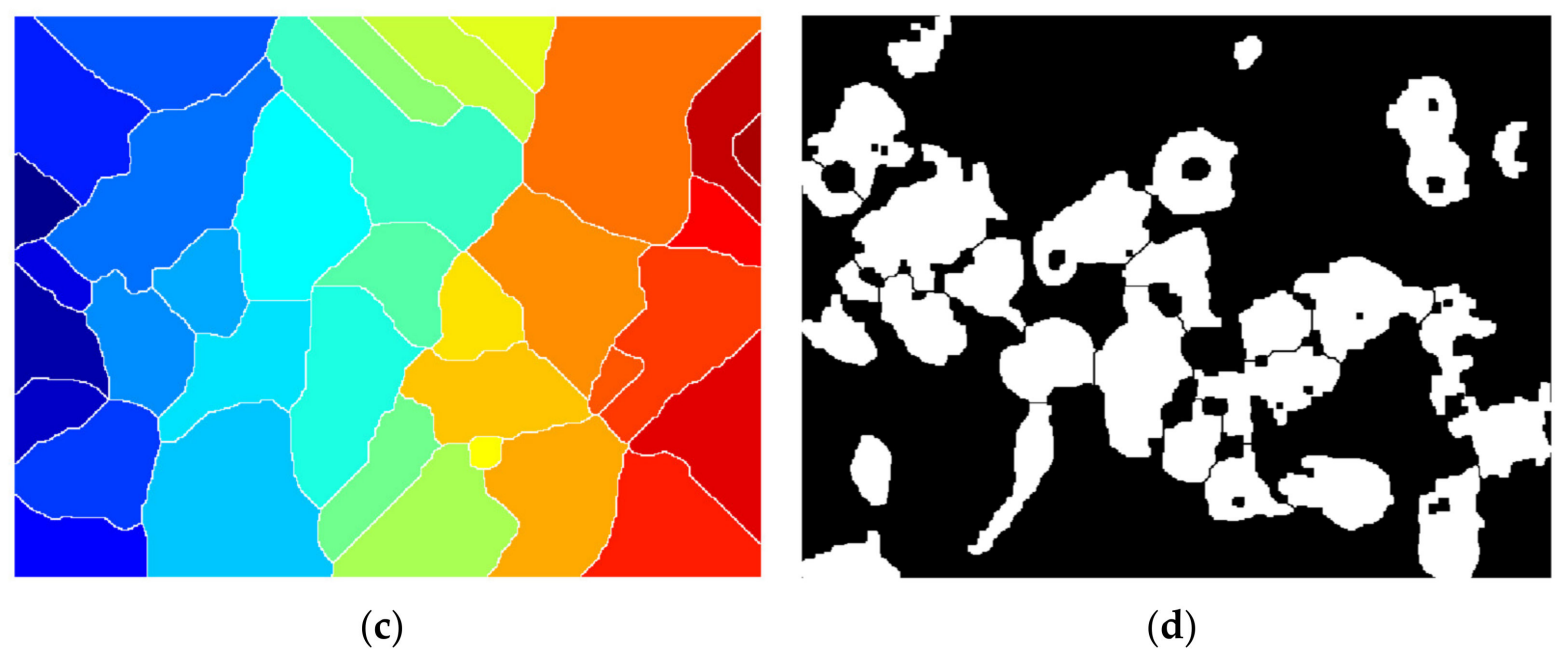
(**a**) Over-segmentation watershed transform; (**b**) nucleus mask over binary image; (**c**) watershed transform with (**b**) imposed; (**d**) segmented image.

Since we have come to the point where each pool represents the cell region connected as a graph, all that is left to do is to finally apply the watershed transform but with the observation that typically over-segmentation (Figure 12a) may appear due to the fact that each and every local minimum is settled to be a catchment region; hence, some parts of cell are segmented inconsequentially. In this direction, a mask of potential nuclei (Figure 12b) is determined using the *imextendedmin* function that computes deep regional minima locations. The minima transform given as an input parameter is 3, and the default 8-pixel connection is used.

The mask is then imposed to force the local minimum basins into those determined nuclei for more accurate splitting, by using the *imimposemin* function (Figure 12c).

Lastly, spots that are less than a 500-pixel threshold are eliminated since they are not acknowledged complete cells (Figure 12d).

### 3.3. Data Quantification

**Figure 13:**
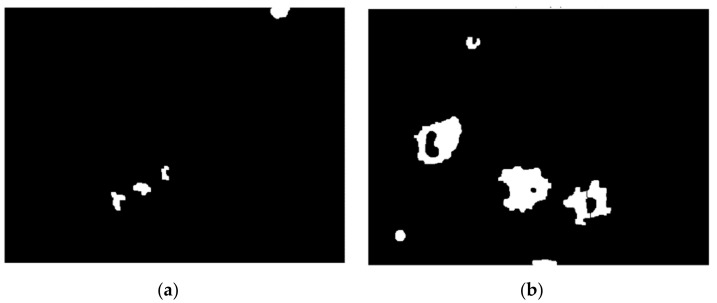

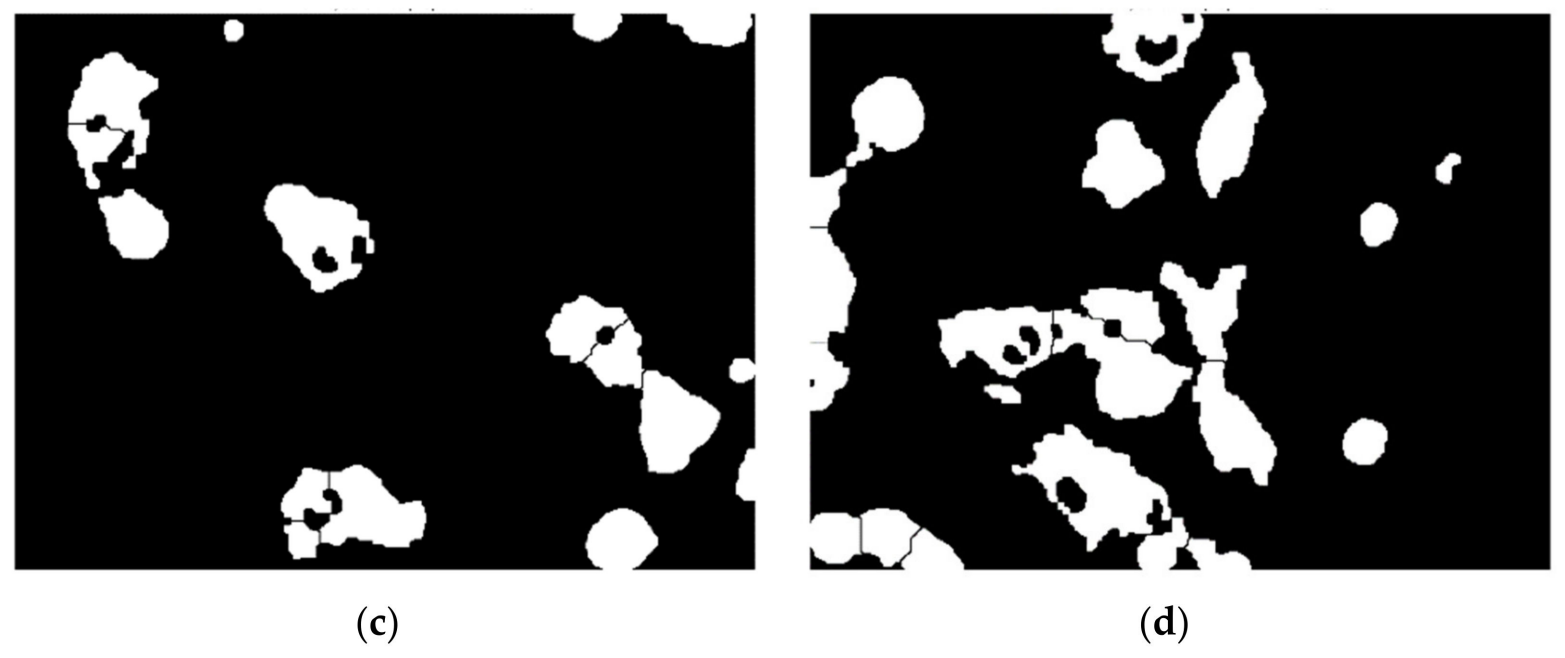
Set of processed images: (**a**) Stage A—control, cells detected: 4, cell area: 0.62%; (**b**) Stage B—12.5 μg/mL, cells detected: 7, cell area: 4.76%; (**c**) Stage C—25 μg/mL, cells detected: 16, cell area: 10.81% (**d**) Stage D—50 μg/mL, cells detected: 22, cell area: 17.75%.

Figure 13 depicts the effect of various dosages on cell culture. Stage A denotes the cells in their initial state, stage B introduces slight amount of treatment, and stages C and D steadily increase the concentration. As can be seen, the higher the concentration, the larger the number of luminous spots, and therefore the treatment’s efficacy is amplified.

**Figure 14:**
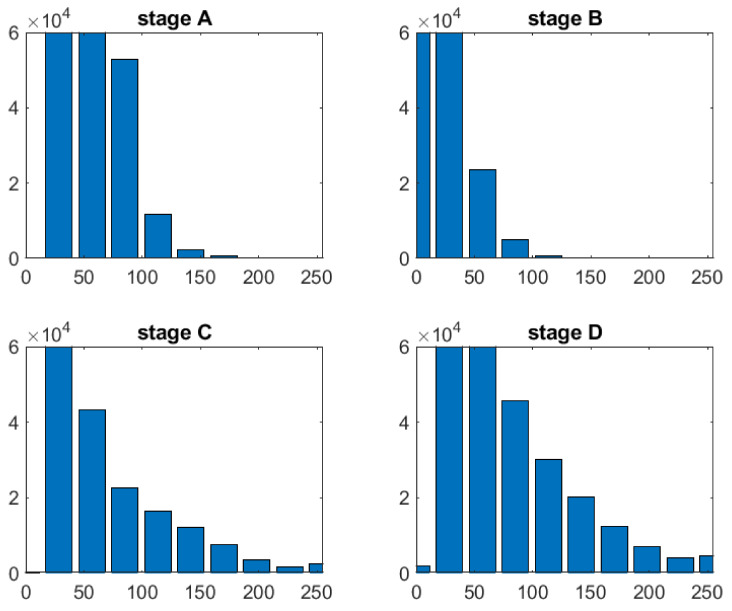
Original images histograms.

Henceforth, the amount of clear, white spots or the covered surface are related directly to the efficacy of each example in the case. Figure 14 contains a processed set and several extracted characteristics for the sampled cell group. After the binary picture has been segmented, *regionprops* may be used to get important cell-related numerical data such as area, perimeter, roundness degree, or extrema locations. Each cell counted in a structural element corresponds to significant data for measuring cell viability and proliferation; their whole surface is computed as a rate across the full picture area to provide a clear contrast from one stage toward another. Molecules have also been seen to form clusters, particularly when subjected to greater concentration levels.

Additionally, after histogram analysis an important threshold value was chosen to quantify all white points within each image, this number being passed as an extracted descriptor that characterizes a specific stage of treatment. The value is expressed however as a percentual proportion relative to the image dimension.

The histograms were produced from original pictures (Figure 14) and showed significant variations across phases, particularly D vs. A, where the number of high-intensity pixels was significantly higher, with values exceeding 200 in the range. Because the black pixels from the dark-field backdrop are prominent in all pictures, the distribution is right-skewed. For a final conclusion, each result from a set of four dose-dependent pictures was compared to phase A, which comprised just noise collected by the microscope equipment and provided no meaningful information. Modifications from the starting point, i.e., stage A, were the focus of attention.

The final output from the processing function is a cell array containing the reference stage for the nanostructured exposure, the percentual area, number of blobs detected, percentual number of white points, the binary image mask of segmented cells expressed as an array, and the structure with the features determined by regional properties function. In the structure, each cell from the specific picture has a corresponding area, filled area, circularity parameter (the more round the shape, the closest to 1 is the value), eccentricity (the measure of ellipsoidal degree, 0 for a circle and 1 for a straight line), maximum feret properties, and minimum feret properties. Each line from the final cell array corresponds to one segmented image.

### 3.4. User Interface

The principal aim is extraction of cell measurements representing the treatment efficiency in therapy of colon and rectum cancerous conditions. For achieving this, it was necessary to construct a standalone application that can permit the user-friendly and intuitive interaction between biomedical specialists and the developed algorithm.

The development environment used was MATLAB App Designer provided by MathWorks. By using this, the design of the interface was created aimed towards general user comfort. The scope was to keep it as simple and intuitive as possible because the user may or may not be a specialist in computer engineering.

**Figure 15:**
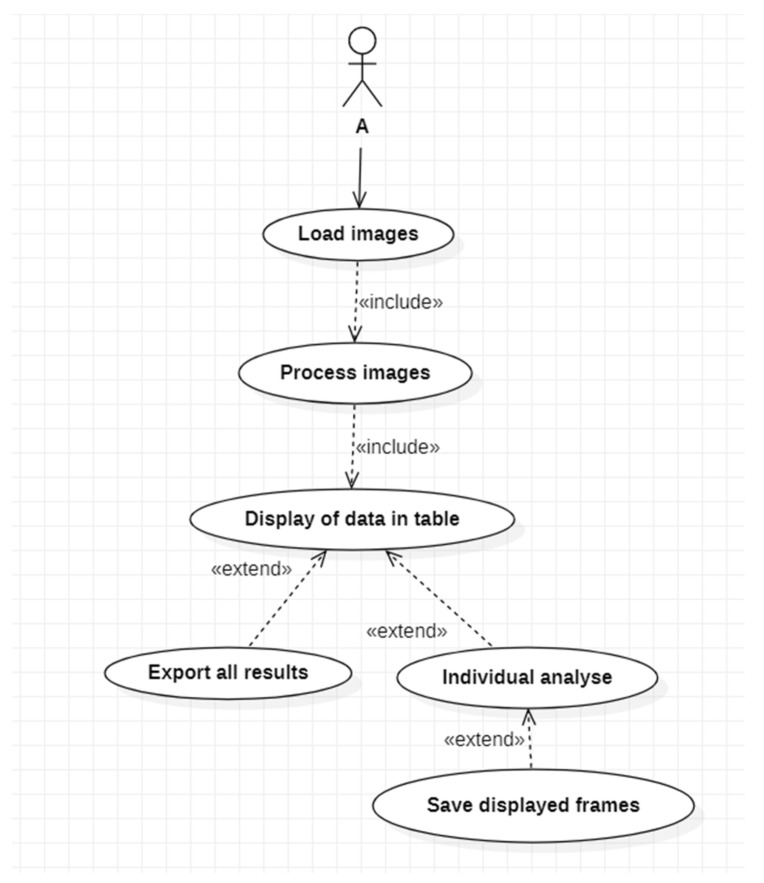
Use case diagram.

Therefore, the principal components of the application—as presented in Figure 15—are as follows:

- Multiple objects of type Button through which images will be selected from a memory location and loaded into the workspace, another one for starting the processing algorithm and a third one that can be used for exporting the extracted data from the application to an external file in any desired location.
- One Image object that permits the visualization of the original loaded images.
- Two objects of type Axes employed for visualization of segmented images and for displaying the image histograms for each selected picture that was processed.
- One object of type Table being exploited to display significant data and features extracted after employing the algorithm on each and every image.
- Labels used for presenting general information.

The most important functions that will manage the flux of data are presented further.

◦ The function SelectimagesButtonPushed() is the one that when the button is actioned, calls for image selection and loading. This opens a new dialog window from where the location and the image or the set of images we desire to process is chosen.
  The process is not automatically started after loading from memory because this could increase the whole operation a lot more and the risk of reading from memory errors can be increased.
◦ The function ProcessimagesButtonPushed() is invoked when the corresponding button is pushed. This will iterate through a loop and call the cell_segmentation() method on every image loaded into the workspace. The data that is extracted from each image is then saved in a cell array that can be used later for retrieving information necessary for table and image displaying.
◦ Cell_segmentation() is the function containing the algorithm developed for segmentation and feature extraction. It is taking as input one image at a time and the corresponding flag depending on the stage of drug treatment that was applied on the cell culture captured.
◦ The function UITableCellSelection() is a callback used in the moment an event of table cell click is encountered; when one of the table cells is activated, the respective image from that particular line will be displayed as original image, segmented image, and histogram representation.
◦ The function ExportdataButtonPushed() responds to the third button event, by opening a new dialog box where the user have to choose the location and the file name in which all data results will be saved as a .mat file. The mat file is preferred in these conditions because as outputs we are interested in different data types such as, image matrices, structures containing labeled images, or tables containing geometrical and structural features for each cell detected in an image. The combination of data types is facile when using cell arrays and mat files that can be easily imported again to MATLAB and further utilized. After the file has been created successfully, a message box will notify the user about the action status.

The showcase in Figure 15 is the use case diagram for application usage. As can be observed, the application has a straightforward flow, allowing the user to choose the method of analyzing and saving the information supplied.

**Figure 16:**
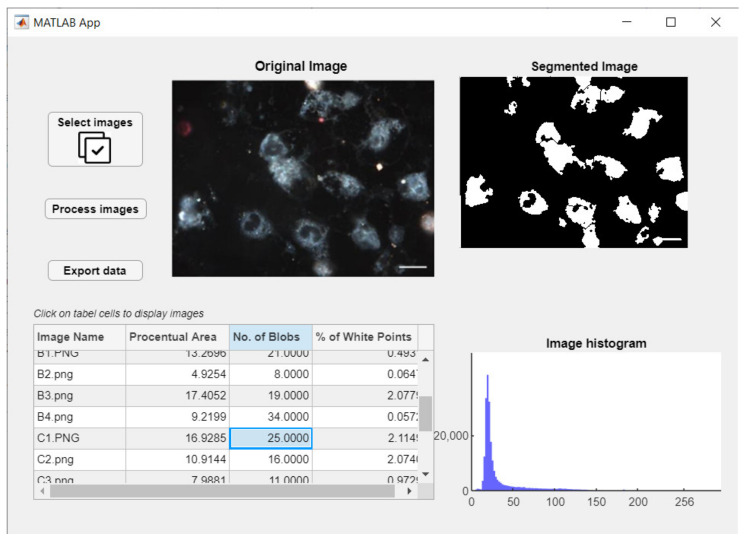
Application runtime exemplified.

The main window will appear after launching the application, as that in Figure 16. Here, the foremost components and functionalities can be easily observed: a button responsible for loading batches of images, another one for starting the image processing function, one for exporting extracted data, and one data table and three visual components for graphical representation of images.

## 4. Discussion

### 4.1. Segmentation and Cell Features

The quantification of single-cell properties is one of the most important resolutions; therefore, it is critical to test the suggested cellular identification method’s correctness. The segmentation results were compared to the measurements using manually counted cells as a reference point. As a consequence, the binary masks generated by the segmentation technique were first determined in order to analyze the findings. After that, human perception was used to assess each outcome. To do this, around 1300 cells were tested using 28 distinct test captures, with roughly 100 individually annotated cells. Error values were obtained for each picture *i* to determine the segmentation accuracy rate. Relation (6) may be used to calculate the error rate, where $B\left(b_{j}^{cell}\right)$ is a binary indicator that accepts 1 or 0 values depending on the mask overlays. If $b_{j}^{cell}$ (mask pixels of cell *j* after applying the suggested technique) are the same as $b_{j}^{cell,Original}$ (mask pixels of cell *j* in the human labeled picture *i*), then $B\left(b_{j}^{cell}\right)=1$, else $B\left(b_{j}^{cell}\right)=0$. The total number of cells in a frame is denoted by *N*.

(6) $$\varepsilon_{i}=\frac{\sum_{j=1}^{N}B\left(b_{j}^{cell}\right)}{N}\times 100\%$$

The calculated error rates show a mean error of 10.6% and a standard deviation of 2.4 percent, which is an excellent result for cell area determination.

**Figure 17:**
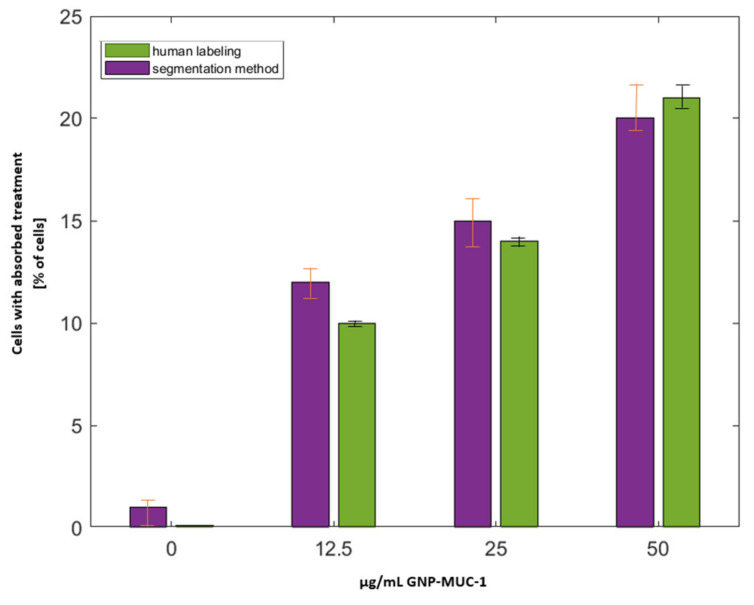
Determined cells in each concentration category manually vs. through the proposed segmentation method.

Each image is assigned to a category based on the treatment concentration (0, 12.5, 25, 50 μg/mL), and the calculated values are stored in a table with four distinct rows, as shown in Figure 17, which indicates a visual representation of the percent number of cells determined by the segmentation method and human labeling.

The mean absolute error (MAE = 0.287) shows that there is no substantial difference between the reference number of cells and the determined cells. As a result, the suggested method may be used to determine single cells in cell populations in the ongoing research.

To determine the cytotoxic effect induced by the newly discovered nano bio-chemical product, the viable cells are measured after exposure. Dead cells are the ones that internalize the compound, so they become fluorescent. Hence, a high number of intense cells detected means a high rate of treatment success. A minimal viability is then determined for higher concentrations applied. On the other hand, control trials (stage A) report insignificant particulate white light reflection points corresponding to gold nanoparticles, such that all images from control phases are considered to contain artifacts (air bubbles from the microscope probe) and are being eliminated as noise.

The image processing time implies complex methods, such that the time necessary until output is in the order of seconds. For a single image, around 36 milliseconds are required to finalize the process tested on an average processor of 2 GHz.

### 4.2. ANFIS (QSAR) Model

The trials are still ongoing, and thus the model is still in its early stages. Internal validation (or cross-validation) and external validation, on the other hand, will be used to validate the models that will have been constructed. The model developed will be used for prediction as well as optimization. The entire data set will be split into training and test sets; however, this is an important stage since it must include all of the information gathered. The training set is used to construct the model, whereas in the test set, the success and consistency of the model in prediction may be determined. There is a variety of ways to think about splitting, each with its own set of benefits and drawbacks.

The goodness of fit parameter characterizes the predictions on the training set. K-fold validation is a cross-validation approach that involves dividing data sets into k distinct subsets and using each one as a testing set one by one. A q^2^ cross-validation coefficient that surpasses a 0.6 criterion is used to evaluate good prediction models. Additionally, with values exceeding 0.6, the squared correlation coefficient R^2^ estimates a reasonable difference between observed and anticipated activity. Both are assessed on internal tests, and the next stage is external validation when these two are satisfied.

External validation, on the other hand, necessitates a precise partition of data into training and testing proportions of 80/70 and 20/30 percent, accordingly. In this case, the aim is to assess how well the model can predict the test set.

The ideal method for evaluating the resulting model would be to use a variety of indicators, such as statistics, prediction accuracy, and domain applicability. Statistical parameters such as correlation coefficient, mean squared error (MSE), and root mean squared error (RMSE) determine the model robustness, yet the level of prediction is not tracked.

Each method has its advantages and drawbacks, such that many things must be considered to avoid overoptimistic results: diversity of data, statistical methods, sizes, and study purpose. By comparing the results from all three different approaches, we can observe the problems in the model structure.

## 5. Conclusions

Predictive models show their benefits in various domains from everyday life or even complex processes that have or have not at a first glance anything in common with technology. In recent years, computer vision evolved at a high rate, resolving easily multidisciplinary problems (medicine, transports, economy, signal processing), especially by using machine determined models to anticipate reactions of dynamic processes using experimental data.

Moreover, imaging has been named one of the greatest achievements of the twentieth century due to its influence on medicine and biology. Medical imaging systems have evolved greatly during the last several decades. Their properties, including sensitivity, resolution, and acquisition speed, have all improved significantly. Biologists may now acquire a picture all of the molecular activity within a tissue via imaging. In biology and medicine, advanced image processing and analysis techniques are widely used. Engineers, biologists, and medical physicists are finding best solutions to improve quality of life by means of new technological discoveries.

Regarding the field of image processing and analysis of medical images, there are two main issues which have to be addressed:

- Improving the image data acquisition quality.
- Robust, efficient, and accurate extraction of information (i.e., feature) from medical picture data.

Image enhancement techniques such as noise filtering, contrast, and edge enhancement fall within the former category, whereas image analysis methods deal primarily with the latter issue. The images generated in medical and biological applications are complex and vary substantially from application to application.

The model’s goal is to determine if vaccination nano-compounds are safe and effective. The image processing step is important for feature extraction, not only in terms of pixel values, but also in terms of independent cell information, in order to estimate the response intensity of various molecules exposed to the therapeutic vaccination. The results gathered are decisive for further investigation. Consequently, the features retrieved are first engaged in the feature optimization process, and then a suitable model is determined using the neural network generated.

The paper presents preliminary results on the development of a model (the QSAR model) that links and predicts the effect of the therapeutic vaccines on cancer treatment, using molecular descriptors. One important step taken into consideration is image processing, in order to extract and measure the vaccine efficiency for further use in the model design. Clinical data sets are used, having ethical approval. The research is opportune for the times we live in, as it integrates concepts from artificial intelligence and nanomedicine for taking one step further in cancer treatment.

The semi-automatic algorithm proposed has the implemented methodology for real-life application, and it offers satisfactory results towards image segmentation and cell determination. A number of molecular descriptors necessary in the regression phase of model development are determined by advanced application. This work tries to find the best solutions to help the medical field and medical workers achieve progress in regard to survival rate and in curing patients that suffer from different forms of cancer. On top of that, through the research, there have also been presented image processing techniques that best suit hyperspectral cell imaging, which is necessary for further data analysis. The technologic approaches that helped in achieving the stated goal are a selection of traditional and new techniques assimilated from the literature and rigorously combined for determining finest feature extraction results. For the resulting artificial intelligence algorithm, human intervention is minimal and is required only for selecting properly some of the parameters used. Further, the only issue a user will have to deal with is the loading of data, through just one click.

Since the error rate for cell area indication is around 10% for a modest set of images comprising nearly 1300 cell identities, the proposed algorithm can be considered as strongly well-adapted, with successful results.

The final product for the time being is a desktop application that facilitates interaction between the user and the algorithm developed in MATLAB^®^ [30]. Through this, the user acquires the images from local memory storage data, and the image processing algorithm embedded automatically outputs descriptive data for further model development. The user has the possibility of analyzing the final image segmentation or to just save the extracted features from the provided images.

These results are strongly related to the authors’ research in the area of cancer diagnosis and prevention [31,32].

The mathematical model for the vaccine will be placed under further development as soon as the whole experimental process is finalized. There is already some amount of characterization data that can be combined in preliminary analyses that will ensure safety and efficiency of treatment.

## Figures and Tables

**Table 1:** Structural descriptors.

Measure	Definition	Circle	Square
Form factor	$\frac{4\pi \times Area}{Perimeter^{2}}$	1	π/4
Roundness	$\frac{4\times Area}{\pi \times MaxDiameter^{2}}$	1	π/2
Aspect ratio	$\frac{MaxDiameter}{MinDiameter}$	1	1
Solidity	$\frac{Area}{ConvexArea}$	1	1
Extent	$\frac{TotalArea}{Area\;Bounding\;Rectangle}$	π/4	1
Compactness	$\frac{\sqrt{\left(4\times Area\right)}/\pi }{MaxDiameter}$	1	$\sqrt{2}/\pi \;$
Convexity	$\frac{ConvexPerimeter}{Perimeter}$	1	1
